# A Loss to the Leeds Infirmary

**Published:** 1913-06-07

**Authors:** 


### A Loss to the Leeds Infirmary.

MR. LITTLEWOOD'S RETIREMENT.

Mr. Harry Littlewood, who has been thirty-four years in practice as a surgeon in Leeds, and has acted as surgeon to Leeds Infirmary, is retiring into private life, and was entertained last week by about two hundred members of the medical profession of Leeds and from many parts of Yorkshire.

Dr. J. E. Eddison proposed the toast of "Our Guest" in a humorous speech, in which he referred to the large series of operations which he had performed. He said the other day Sir Berkeley Moynihan was referred to as to the large number of operations that took place in the Far West, but these were few compared with those that had been performed by Mr. Littlewood. He had devoted his attention in particular to the lower intestines, and had brought to his work an extraordinary degree of skill, and had been financially successful in his profession. He had prescribed poisons, more or less dangerous, and he had given advice more or less "reasonable." (Laughter.)

Mr. Littlewood said, in response, he was overwhelmed by their kindness, and warmly thanked them. He recalled many reminiscences in connection with his past experience amongst them, and said he should always treasure tender memories of Leeds and its infirmary. Mr. Littlewood has been presented with a silver cigar case from the medical school students, and on leaving twelve students withdrew the carriage horses.

